# Treatment of urinary tract infections in Swiss primary care: quality and determinants of antibiotic prescribing

**DOI:** 10.1186/s12875-020-01201-1

**Published:** 2020-07-01

**Authors:** Andreas Plate, Andreas Kronenberg, Martin Risch, Yolanda Mueller, Stefania Di Gangi, Thomas Rosemann, Oliver Senn

**Affiliations:** 1 Institute of Primary Care, University and University Hospital of Zurich, Pestalozzistrasse 24, 8071 Zurich, Switzerland; 2 Swiss Center for Antibiotic Resistance, Institute for Infectious Diseases, University Bern, Bern and Medix General Practice Network, Bern, Switzerland; 3 labormedizinisches zentrum Dr Risch Ostschweiz AG, Buchs, St. Gallen, Switzerland; 4 Department of Family Medicine, Center for Primary Care and Public Health (Unisanté), University of Lausanne, Lausanne, Switzerland

**Keywords:** Urinary tract infection, Antibiotic prescribing quality, Primary care, Switzerland, Quality indicator

## Abstract

**Background:**

Urinary tract infections are one of the most common reasons for prescribing antibiotics in primary care. Current guidelines recommend fosfomycin, nitrofurantoin, or trimethoprim - sulfamethoxazol as empiric first line antimicrobial agents in uncomplicated infections. However, there is evidence that the use of fluoroquinolones, which are no longer recommended, is still inappropriate high. We determined antibiotic prescription patterns, quality and factors affecting antibiotic prescriptions in urinary tract infections in primary care in Switzerland.

**Methods:**

From June 2017 to August 2018, we conducted a cross-sectional study in patients suffering from a urinary tract infection (UTI). Patient and general practitioners characteristics as well as antibiotic prescribing patterns were analysed.

**Results:**

Antibiotic prescribing patterns in 1.352 consecutively recruited patients, treated in 163 practices could be analysed. In 950 (84.7%) patients with an uncomplicated UTI the prescriptions were according to current guidelines and therefore rated as appropriate. Fluoroquinolones were prescribed in 13.8% and therefore rated as inappropriate. In multivariable analysis, the age of the general practitioner was associated with increasing odds of prescribing a not guideline recommended antibiotic therapy.

**Conclusions:**

We found a high degree of guideline conform antibiotic prescriptions in patients with an uncomplicated urinary tract infection in primary care in Switzerland. However, there is still a substantial use of fluoroquinolones in empiric therapy.

## Background

Antibiotic overuse and inappropriate use are associated with the emergence of resistant pathogens [1]. The majority of antibiotics are prescribed in ambulatory care [2] and urinary tract infections (UTI) are beside respiratory tract infections one of the most common reasons for prescribing antibiotics [3]. Many current guidelines (for example Swiss national guidelines or United States IDSA guidelines) [4–6] recommend the use of nitrofurantoin, fosfomycin and trimethoprim -sulfamethoxazol (TMP/SMX) as empiric first line antimicrobial agents for the treatment of an uncomplicated UTI (uUTI). TMP/SMX is recommended when the local resistance patterns of *Escherichia coli* (*E. coli*) are known and the estimated susceptibility is over 80%. Despite the fact, that the overall usage of antibiotics in Switzerland is low compared to other European countries [7], there is evidence, that there is a significant amount of inappropriate antibiotic prescriptions in UTI [8]. Especially fluoroquinolones (FC) were still prescribed. Despite their undisputed efficacy in the treatment of UTI, their excessive use in the last decades resulted in raising resistances not only in uropathogens, but also in pathogens causing extra-urogenital infections [5]. Due to their importance in the treatment in extra-urogenital infections, the use of FC as an empiric first line antimicrobial agent in uUTI is no longer recommended [5, 6, 9]. Furthermore, FC use is associated with further serious adverse effects (for example tendinitis and tendon rupture, but also prolonged QTc, or *Clostridium difficile* associated diarrhoea) and both the US Food and Drug Administration and the European Medicines Agency released warnings and called for a more restrictive FC use [10, 11].

In this study, we determined the prescribing patterns and the quality of antibiotic prescribing in ambulatory UTI and report factors associated with appropriate or inappropriate antibiotic prescribing.

## Methods

We conducted a cross-sectional study between June 2017 and August 2018 in primary care practices in Switzerland to evaluate resistance patterns as well as treatment patterns in patients with lower UTI (cystitis). Details of the study design, as well as recruitment procedure are reported elsewhere [12]. In this study, lower UTI was defined as the new onset of typical symptoms (dysuria, pollakiuria, urgency or haematuria) in combination with a positive urine dipstick (positive leucocytes). All UTI in otherwise healthy women without the history or the clinical suspicion of any functional or anatomical abnormalities of the urinary tract were considered as uncomplicated. All other conditions as well as UTI in men were considered as complicated. One hundred sixty-one Swiss primary care practices as well as two large “walk-in” practices (larger practices with the following characteristics: longer opening hours, 7 days a week open, offering in-hours continuity of care and out of hours care for self-referred patients (walk-ins) without having a previous appointment) participated in the study. In addition to microbiological work-up, treatment information of the general practitioners (GP) were obtained. Empirical treatment of a uUTI with fosfomycin, nitrofurantoin or TMP/SMX were considered as adequate according to national guidelines [4]. Although an antibiotic therapy is recommended, therapeutic approaches with a standby treatment, delayed prescriptions, and even withholding antibiotic treatment and symptomatic therapy were rated as suitable options in uUTI and considered as a guideline adherent treatment in all of our calculations [4]. Overall quality of antibiotic prescribing was determined according to the European Surveillance of Antimicrobial Consumption (ESAC) disease-specific quality indicators for outpatient antibiotic prescribing [13].

### Statistics

Summary statistics were reported as means (standard deviation, SD), and number (percentage, %) as appropriate. Antibiotic prescription patterns were compared between complicated UTI (cUTI) and uUTI using independent sample Student’s t test and chi-square or Fisher’s test, as appropriate. Prescription of FC means prescription of at least one out of ciprofloxacin, levofloxacin, moxifloxacin, or norfloxacin. Univariable and multivariable regression analyses were performed to assess GP and patient determinants associated with the quality of antibiotic prescribing. The results of the “walk-in” clinic were only excluded when analyzing GP characteristics in univariable and multivariable regression analyses, due to the fact that different GPs were on duty and we could not match prescribing patterns with a single physician. In 13 patients, final diagnosis (cUTI or uUTI) was missing; hence, these patients were excluded for regression analysis, but still included in the overall analysis. Multilevel logistic models, with GP as first level clustering variable, were performed for the prescription of guideline antibiotics or for guideline adherent treatment of uUTI, versus any other antibiotics, and separately, for the prescription of fluoroquinolone versus guideline antibiotics. The mixed models with random effects (GP), were specified as follows: “Antibiotic prescription / treatment ~ Fixed Effects (X) + random effects of intercept (GP)” where X = GP characteristics: sex, age, years of experience in practice, type and location of practice, work time percentage, affiliation to a medical network, language (Latin: Italian or French vs. German speaking); patient characteristics: age, sex, history of UTI, antibiotic exposure within the last 3 months, inpatient treatment within the last 6 months, travel history within the last 12 months, reason for the initial encounter, type of UTI. In univariable analysis, every fixed effect (X) was considered separately in a single model. In multivariable analysis, instead, relevant effects, that is predictors with *P* ≤ 0.2 in univariable analysis, were considered together. Stepwise backward elimination was used to develop final multivariable models with best fit for the outcome of interest. Results of regression analyses were presented as odds ratio (OR) (95% confidence interval (CI)). ICC was also reported in multivariable analysis. For all tests, *P* ≤ 0.05 was considered statistically significant. According to ESAC [13], we defined the acceptable ranges of receiving a recommended antimicrobial agent (between 80 and 100%), as well as the use of a FC antimicrobial agent (< 5%). All analyses were carried out using statistical package R (https://www.R-project.org).

## Results

One thousand three hundred fifty-two patients were recruited by 161 GPs and the two large “walk-in” practices. Basic characteristics of the conducting GPs are described in Table 1. 1210 (90.4%) patients had a diagnosis of a uUTI and 129 (9.6%) of a cUTI**.** 94.9% of the patients were female and the mean age was 54 years **(**Table 2**)**.

**Table 1:** Basic characteristics of 161 participating General practitioners

	N (%)
Sex	
Male	112 (69.6)
Female	49 (30.4)
Age (mean, SD) (missing *n* = 6)	52.18 (8.83)
Years of experience in ambulatory care (mean, SD) (missing *n* = 11)	14.97 (9.56)
Practice type (missing *n* = 2)	
Single practice	42 (26.6)
Double practice	41 (25.9)
Group practice (≥3 GPs/practice)	75 (47.5)
Work-time % of GP (missing *n* = 3)	
30–60%	36 (22.9)
70–80%	39 (24.8)
> 80%	82 (52.2)
Affiliation to a GP network (missing *n* = 2)	
Yes	105 (66.5)
No	53 (33.5)
Language of GP	
Latin (Italian / French)	25 (15.5)
German	136 (84.5)
Location of GP^a^	
Central Switzerland	18 (11.0)
Eastern Switzerland	39 (23.9)
Midlands	31 (19.0)
Northwestern Switzerland	30 (18.4)
Lake Geneva region	15 (9.2)
Ticino	5 (3.1)
Zurich	25 (15.3)

Data shown as absolute numbers and in percentage (in parenthesis) if not stated else. SD: standard deviation; GP: General practitioner

^a^contains geographical information of the 161 GP practices plus the two walk-in practices

**Table 2:** Patient characteristics and antibiotic prescription patterns

	Total	Uncomplicated UTI	Complicated UTI	P
**Patient characteristics**				
N	1352	1210	129	
Sex				
M	69 (5.1)	0 (0)	69 (53.5)	< 0.001
F	1283 (94.9)	1210 (100)	60 (46.5)	
Age (mean, (sd))	53.75 (20.83)	53.16 (20.88)	58.75 (19.90)	0.004
Antibiotic therapy				
No AB	103 (7.6)	88 (7.3)	10 (7.8)	
One AB	1241 (91.8)	1116 (92.2)	118 (91.5)	0.921
Two AB	7 (0.5)	5 (0.4)	1 (0.8)	
> 2 AB	1 (0.1)	1 (0.1)	0 (0.0)	
**Antibiotic prescription patterns - Recommended first line antibiotics**				
Nitrofurantoin	180 (14.4)	163 (14.5)	16 (13.4)	0.855
Fosfomycin	519 (41.6)	501 (44.7)	17 (14.3)	< 0.001
TMP / SMX	311 (24.9)	289 (25.8)	18 (15.1)	0.015
**Antibiotic prescription patterns - Quinolone antibiotics**				
Ciprofloxacin	121 (9.7)	67 (6.0)	53 (44.5)	< 0.001
Levofloxacin	2 (0.2)	2 (0.2)	0 (0.0)	–
Moxifloxacin	3 (0.2)	3 (0.3)	0 (0.0)	–
Norfloxacin	93 (7.4)	84 (7.5)	8 (6.7)	0.906
**Antibiotic prescription patterns - others**				
Amoxicillin	14 (1.1)	11 (1.0)	3 (2.5)	0.291
Amoxicillin/Clav	5 (0.4)	4 (0.4)	1 (0.8)	0.975
Cefuroxim	3 (0.2)	2 (0.2)	1 (0.8)	0.677
Other*	6 (0.5)	3 (0.3)	3 (2.5)	0.007

*N* number of patients; *M* male; *f* female; *sd* standard deviation; *AB* antimicrobial agent; *UTI* urinary tract infection; *TMP/SMX* trimethoprim - sulfamethoxazol; *Clav* clavulanic acid

*: This group consist of ceftriaxon (1), azythromicin (1), metronidazol (2), fluconazol (1), and unknown (1)

### Antibiotic prescribing patterns

**Fig. 1:**
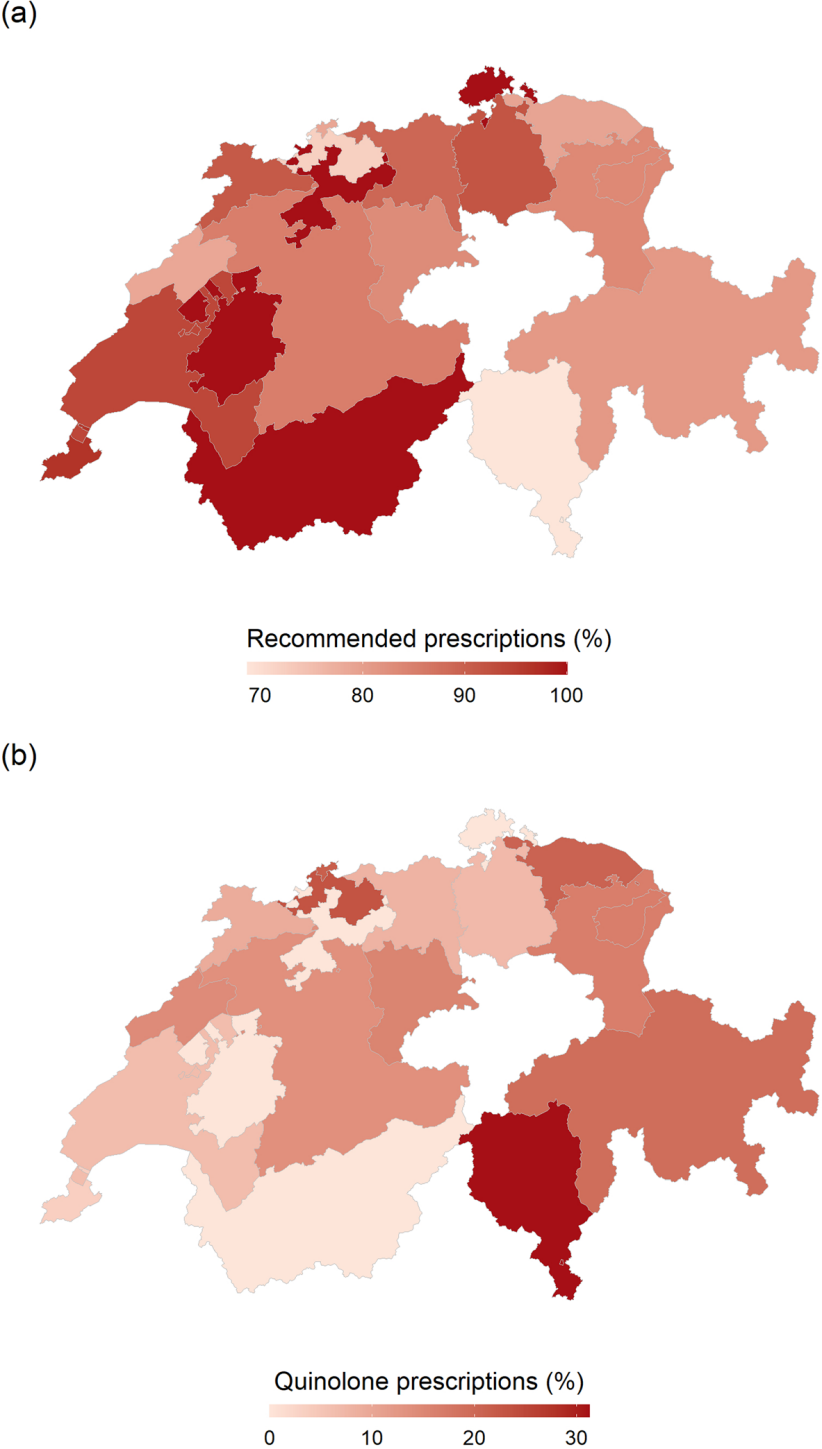
Antimicrobial prescribing patterns in Switzerland

In total 1241 (91.8%) patients were treated with one antimicrobial agent, 8 patients (0.6%) with two or more antimicrobial agents. One hundred three patients (7.6%) did not receive any antibiotic. Of these, 47.6% received at least one drug for symptomatic therapy, mainly phytotherapeutics (*n* = 34) or analgesic drugs (*n* = 22). Regarding the choice of antimicrobial agents, fosfomycin (44.7%), TMP/SMX (25.8%), and nitrofurantoin (14.5%) were most often prescribed in uUTI, resulting in a guideline conform empiric antibiotic therapy in 84.7%. Including patients with standby treatment and no antibiotic treatment, we found a guideline adherent approach in 85.8% in uUTI. According to the ESAC definitions for treatment in uUTI, the overall percentage of females receiving any systemic antibiotic therapy (92.7%) and the percentage of receiving a recommended antibiotic therapy (84.7%) were within the desired range. In contrast, the prescription rate of FC in all uUTI patients (13.8%) was above the desired range (< 5%), resulting in an evidence-performance gap of 8.8% (Table 3). The highest amount of guideline recommended antibiotic prescriptions was seen in the Geneva region (96.6%), the lowest in the canton of Ticino (64.7%) (Fig. 1 & Supplemental Table 1).

**Table 3:** Prescribing performance according to disease specific antibiotic prescribing quality indicators in Europe

Reference No (according to 13)	Title	Acceptable Range (%)	N (%)
3a.	Percentage of female patients older than 18 years with cystitis / other urinary infection (ICPC-2-R: U71) prescribed antibacterials for systemic use: ATC: J01	80–100	1122 (92.7%) (ref pop. *n* = 1210)
3b.	receiving the recommended antibacterials: ATC: J01XE or J01EA or J01XX	80–100	950 (84.7%) (ref pop. *n* = 1122)
3c.	receiving fluoroquinolones: ATC: J01M	0–5	155 (13.8%) (ref pop *n* = 1122)

*N* number of patients; *ref. pop* reference population

Panel part A shows the relative prescriptions rates of recommended antimicrobials in female patients with an uncomplicated urinary tract infection in Switzerland. Panel part B shows the relative prescriptions rates of Quinolone prescriptions.

In patients with cUTI 92,3% were treated with antibiotics, most often ciprofloxacin (44.5%), TMP/SMX (15.1%) and fosfomycin (14.3%) were prescribed. Ciprofloxacin was more often prescribed in cUTI compared to uUTI (44.5% vs. 6.0%, *p* = < 0.001). In contrast, fosfomycin and TMP/SMX were more often prescribed in uUTI (44.7% vs. 14.3%, *p* < 0.001, and 25.8% vs. 15.1%, *p* = 0.015, respectively) **(**Table 2**)**.

### Determinants of antibiotic prescribing

Results of uni- and multivariable analyses for guideline recommended treatments of uUTI are reported in Table 4. In multivariable analysis, only increasing age of the GP was significantly associated with reduced odds of prescribing any guideline recommended antibiotic treatment (OR: 0.96, *p* = 0.010) or a guideline adherent treatment (OR: 0.96, *p* = 0.012).

**Table 4:** Logistic regression analysis for guideline recommended and guideline adherent antibiotic prescriptions in uUTI

		**Guideline recommended**^**a**^**therapy**					**Guideline adherent**^**b**^**therapy**			
		**Univariable analysis**		**Multivariable analysis*N*** **= 1002, GP = 145, ICC = 0.29**			**Univariable analysis**		**Multivariable analysis*N*** **= 1095, GP = 154, ICC = 0.31**	
**GP characteristics**	**N, GP**	**OR (95% CI)**	***p*-value**	**OR (95% CI)**	**p-value**	**N, GP**	**OR (95% CI)**	**p-value**	**OR (95% CI)**	**p-value**
Sex of GP = female	1067, 156	1.28 (0.67, 2.42)	0.458			1155, 161	1.22 (0.64, 2.34)	0.549		
Age of GP	1023, 150	**0.95 (0.92, 0.98)**	**0.003**	**0.96 (0.93, 0.99)**	**0.010**	1107, 155	**0.95 (0.92, 0.98)**	**0.005**	**0.96 (0.92, 0.99)**	**0.012**
Years of GP experience in practice	991, 146	**0.96 (0.93, 0.99)**	**0.023**			1066,150	**0.96 (0.94, 0.996)**	**0.028**		
Practice type (ref = Single)	1052, 154									
Double		2.06 (0.93, 4.54)	0.073			1139, 159	2.13 (0.96, 4.75)	0.064		
Group		**2.00 (1.02, 3.90)**	**0.044**				1.93 (0.98, 3.81)	0.059		
Work-time % (ref = < 60%)										
61–80%	1046, 153	1.05 (0.44, 2.51)	0.919			1133, 158	1.06 (0.44, 2.57)	0.894		
81–100%		**0.43 (0.21, 0.90)**	**0.026**				**0.44 (0.21, 0.92)**	**0.029**		
Affiliated to a medical network	1052, 154	1.16 (0.62, 2.17)	0.633			1139, 159	1.10 (0.58, 2.06)	0.772		
Language of GP (ref = German)										
Latin	1067, 156	1.18 (0.50, 2.78)	0.703			1155, 161	1.10 (0.46, 2.63)	0.823		
Location of GP^c^ (ref = Other regions)										
Region lemanique	1067, 156	2.58 (0.78, 8.54)	0.120	2.30 (0.71, 7.43)	0.165	1155,161	2.50 (0.74, 8.39)	0.139	2.29 (0.69, 7.57)	0.176
Ticino		0.34 (0.06, 1.98)	0.228	–			0.32 (0.05, 1.93)	0.214	0.36 (0.06, 2.06)	0.249
Zürich		**2.65 (1.08, 6.51)**	**0.033**	2.19 (0.87, 5.48)	0.095		**2.78 (1.13, 6.85)**	**0.026**	2.33 (0.92, 5.92)	0.074
**Patient characteristics**	**N**					**N**				
Previous urinary tract infection	1114	1.22 (0.79, 1.84)	0.362			1202	1.22 (0.79, 1.83)	0.361		
Antibiotics in the last 3 months	1112	0.75 (0.53, 1.09)	0.125			1198	0.74 (0.52, 1.07)	0.101	0.71 (0.46, 1.10)	0.123
Treatment in the last 6 months	1117	0.84 (0.51, 1.46)	0.518			1204	0.83 (0.51, 1.44)	0.498		
Travel history within the last 12 months	1100	**1.59 (1.14, 2.22)**	**0.006**	1.30 (0.88, 1.93)	0.187	1187	**1.59 (1.15, 2.21)**	**0.005**		
Travel within Europe	1100	**1.64 (1.17, 2.29)**	**0.004**			1187	**1.62 (1.17, 2.26)**	**0.004**		
Travel to Asia	1100	0.84 (0.47, 1.64)	0.591			1187	0.93 (0.52, 1.80)	0.826		
Travel to Africa	1100	2.07 (0.73, 8.66)	0.232			1187	2.31 (0.83, 9.6)	0.167		
Travel to Nord-America	1100	1.41 (0.60, 4.15)	0.473			1187	1.37 (0.58, 4.00)	0.516		
Travel to South-America	1100	0.80 (0.35, 2.17)	0.626			1187	0.73 (0.32, 1.99)	0.502		
Initial encounter not because symptoms suspicious for an UTI	1115	1.23 (0.65, 2.59)	0.554			1202	1.36 (0.72, 2.85)	0.369		

^a^Treatment with fosfomycin, TMP-SMX, or nitrofurantoin were rated as guideline recommended

^b^This group consists all patients with a recommended treatment or standby treatment, delayed prescriptions, withholding antibiotic treatment, or symptomatic therapy

^c^Locations of GP different from Zürich, Region lemanique and Ticino was grouped into “Other regions” and defined as the reference category

*n* number of patients; *ref.* reference, *GP* general practitioner: *ICC* Intraclass Correlation Coefficient; *OR* odds ratio; *CI* confidence intervals; *TMP/SMX* trimethoprim / sulfamethoxazole

Including all UTI patients, the diagnosis of a cUTI (OR: 0.12, *p* < 0.001) and the sex of the patient (male) (OR: 0.36, *p* = 0.048) were additionally identified as significant determinants associated with reduced odds of prescribing fosfomycin, nitrofurantoin or TMP/SMX. In addition, the age of the GP and the diagnosis of a cUTI were significant associated with increased odds (OR: 1.04, *p* = 0.033, and OR: 8.95, p < 0.001, respectively) of prescribing any FC antibiotic in multivariable analysis. Particular noteworthy are the relative high intra-cluster correlation coefficient (ICC) in multivariable analysis (0.34, and 0.41, respectively) (Supplemental Table 2a). Differentiating antibiotic prescribing patterns between patients with a uUTI and cUTI, a female gender of the GP (OR: 7.08, *p* = 0.008) in cUTI and increasing age of the GP in uUTI (OR: 1.06, *p* = 0.003) were associated with higher odds of prescribing a FC in multivariable analysis (Supplemental Table 2b). Analysing determinants for each prescribed antibiotic separately showed results that are more heterogeneous and were provided in Supplemental Table 2c.

### Prescribing no antibiotic

Regarding the patients without any antibiotic treatment, multivariable regression analysis identified the age of the GP (OR: 1.04, *p* = 0.036) and the circumstance, that the reason for the initial encounter was not the suspicion for an UTI (OR: 2.97, *p* = 0.002), as determinants associated with higher odds for withhold of a recommended antibiotic therapy (Supplemental Table 2d).

## Discussion

In this study, we determined antibiotic prescribing patterns of general practitioners and factors associated with antibiotic prescribing in patients with an UTI in primary care. We found a high adherence to current national and international guidelines in the empirical therapy in uUTI.

Antibiotic prescribing quality in patients with an UTI differs throughout the European countries. Whereas Sweden or the Netherlands reported high rates of antibiotic prescriptions according to national guidelines and a low use of FC (3, and 7.4%, respectively) [14], every second patient in Hungary was treated with a FC [15]. Recently, Glinz et al. evaluated the quality of antibiotic prescribing in primary care in Switzerland in a nationwide survey for common infectious diseases. They reported inadequate prescribing in 47.3% of all UTI cases, with a high proportion of FC use (37.2%) [8]. However, these data are biased as they represent a subsample of GPs, which belong to “high prescribers” of a nationwide interventional study. In our study, 84.7% of the prescribed antibiotic therapies in uUTI were appropriate. Thus, empiric antimicrobial prescribing quality was within the targeted range of > 80% correct prescriptions according to ESAC. Nevertheless, prescriptions of FC were still inadequate high and did clearly exceed the acceptable range of 5% [13]. Increasing age of the GP and the time in practice were associated with decreased odds of prescribing a guideline recommended or adherent therapy and with increased odds of prescribing any FC antibiotic in most of our calculations. As FC were recommended for a long time [16] many physicians were used to treat an UTI with FC [17]. However, due to the high collateral damage, guidelines changed and FC were no longer recommended. In the treatment of cardiovascular diseases one could show, that the age of the physician was clearly associated with the knowledge level of clinical guidelines and younger GPs showed a significant higher knowledge of the current guidelines [18]. Although comparative data for the treatment of UTI are missing, the assumption, that some GPs treat UTIs “as always” seem to be feasible and could explain, at least in part, our finding. However, due to the still favourable resistance patterns of uropathogenic *E.coli* in Swiss primary care [4, 12], there is no obvious need for the use of FC in the empirical therapy of uUTI. The still high evidence - performance gap of 8.8% in prescribing FC antibiotics, should be addressed by specific interventions. One have to keep in mind, that antibiotic prescriptions are a complex process influenced by many factors (for example personality characteristics, or the interpersonal relation within the patient-GP encounter, as well as external factors like public campaigns and characteristics of the national health care system) [19–22], and previous national studies using health claims data or selective interventions showed no or only limited effects on appropriate prescribing [23, 24]. This highlights the need for multifaceted interventions affecting GPs prescribing decisions as changing prescribing behavior seems possible [25]. A continuous postgraduate education consisting of the provision of Up-to-date clinical guidelines and communication skills training are essential, but need to be embedded in further interventions, for example selective antimicrobial resistance reporting [26, 27], and patient education campaigns.

In contrast to uUTI there is no common consensus on the definition of cUTI [28]. A variety of underlying diseases or host conditions are known to be a risk factor for a cUTI [6] and hence no guidelines with an universal approach to this patient group exist and treatment recommendations, especially for men, differ within the available guidelines [4, 6, 29]. Reports, that for example cUTI in men can be uncomplicated illustrate, that an individual approach, dependent of the underlying disease and local resistance rates, might be necessary [6, 28, 30]. Due to the heterogeneity and the selective lack of guidelines, it is difficult to rate the appropriateness of antibiotic prescribing in this group. We found that the diagnosis of a cUTI or a male gender of the patient were significantly associated with increased odds of prescribing a FC or decreased odds of prescribing antibiotics recommended for uUTI. However, in men it is believed, that an isolated infection of the bladder without an infection of the prostate is rare, so prostate uptake of the antimicrobial agent is an important issue. The common practice, beginning an empiric therapy with an intravenous therapy (for example with a third generation cephalosporin) and switching to an oral agent with a good bioavailability when antimicrobial resistance patterns are available, is not feasible in most cases in primary care, especially if disease severity favours an outpatient treatment with only an oral antimicrobial agent. This point up the importance of a urine culture in cUTI, as the empirical therapy with a FC or TMP/SMX (as both show a good prostate penetration) can be adjusted in case of resistant pathogens. Furthermore, it highlights the need for further research in this field, as current guidelines did not cover well this common issue in ambulatory care.

It is worth mentioning, that we observed in all of our calculations a major ICC. Thus, a quite large amount of variation in prescribing patterns in the individual GP must be due to additional (unmeasured) GP, practice, or patient characteristics. Patient and/or GP preferences, prior microbial results, patient allergies, (local) guidelines, or even missing guidelines in the case of cUTI, might be possible factors.

A significant proportion of the patients was not treated with any antimicrobial agent. The use of anti-inflammatory and/or analgesic drugs [31, 32], or to postpone the use of antibiotics could be another reliable option in selected patients [33]. A recent study reported that two thirds of women seeking care for an UTI episode are willing to postpone antibiotics. Moreover, half of the women waited a couple of days before seeking care in respect to the benign cause of most uUTI episodes [33]. In contrast, Gharbi et al. showed in a recent study, that elderly patients with no or delayed antibiotic prescriptions for UTI are at a significant higher risk for bloodstream infections [34]. Thus, these approaches should be avoided in this patient group. Taken together, treatment options other than immediate antibiotic prescriptions can be a feasible alternative and should be taken into account in ambulatory care and antibiotic stewardship programs.

This study has some limitations. First, we are unaware of the reason regarding the choice of antimicrobial therapy. The personal history of a resistant microorganism, or an allergy or intolerance to a first line antibiotic are potential reasons for the use of an alternative antimicrobial agent. However, although it is unlikely that these factors make a treatment with any of the first line antibiotics impossible, we cannot rule out, that the choice of another antimicrobial agent than the first line antibiotics was the correct clinical approach in some cases. Second, we are unaware of coexisting complaints of the patients at the time of encounter. Although rare, a second infection, which need an antibiotic coverage, could influence the GPs decision of the antibiotic regime towards a FC.

## Conclusions

We could show that there is a very high degree of guideline recommended antibiotic use in the empiric therapy of uUTI in primary care in Switzerland. However, the use of FC in the empirical therapy of uUTI is still inadequate high and should consequently be addressed by multifaceted interventions.

## Supplementary information

**Additional file 1.** Table 1 – Overview of prescribed antibiotics stratified by region. Table 2a – Logistic regression analysis for antibiotics prescriptions. Table 2b: Regression analysis identifying GP and patient characteristics as predictor for prescribing any quinolone antibiotic (vs. recommended). Table 2c: Regression analysis identifying GP and patient characteristics affecting GP antibiotic prescribing patterns. Table 2d: Logistic regression analysis for no antibiotics prescription.

## Data Availability

The datasets used and/or analysed during the current study are available from the corresponding author on reasonable request.

## Abbreviations

CI: Confidence interval
ESAC: European Surveillance of Antimicrobial Consumption
FC: Fluoroquinolones
GP: General practitioner
ICC: Intra-cluster correlation coefficient
OR: Odds ratio
TMP/SMX: Trimethoprim -sulfamethoxazol
c / u UTI: Complicated / uncomplicated urinary tract infection
