# Supplementary material for: Treatment of urinary tract infections in Swiss primary care: quality and determinants of antibiotic prescribing
Source: BMC Fam Pract. 2020 Jul 1;21:125. doi: 10.1186/s12875-020-01201-1 (PMC7329427; doi:10.1186/s12875-020-01201-1)
Supplement: Supplementary file 1 — Additional file 1. Table 1 – Overview of prescribed antibiotics stratified by region. Table 2a – Logistic regression analysis for antibiotics prescriptions. Table 2b: Regression analysis identifying GP and patient characteristics as predictor for prescribing any quinolone antibiotic (vs. recommended). Table 2c: Regression analysis identifying GP and patient characteristics affecting GP antibiotic prescribing patterns. Table 2d: Logistic regression analysis for no antibiotics prescription. [file 12875_2020_1201_MOESM1_ESM.docx]

Supplemental table 1 – Overview of prescribed antibiotics stratified by region^+^.

|  | Recommended antibiotic * | Other antibiotic | No antibiotic |
| --- | --- | --- | --- |
| Switzerland Central-East, n=136 | 107 (78.7) | 22 (16.2) | 7 (5.1) |
| Switzerland Central-West, n=207 | 166 (80.2) | 26 (12.6) | 15 (7.2) |
| Switzerland East, n= 220 | 164 (74.5) | 33 (15.0) | 23 (10.5) |
| Switzerland Genève area, n=29 | 28 (96.6) | 1 (3.4) | 0 (0.0) |
| Switzerland North-East, n=206 | 169 (82.0) | 18 (8.7) | 19 (9.2) |
| Switzerland North-West, n= 268 | 195 (72.8) | 55 (20.5) | 18 (6.7) |
| Switzerland South, n=17 | 11 (64.7) | 5 (29.4) | 1 (5.9) |
| Switzerland West, n= 127 | 110 (86.6) | 12 (9.4) | 5 (3.9) |

Only patients with uUTI included. Data shown as absolute numbers and percentage (in parentheses).

^+^: Regions were selected according to the regions defined by Swiss Centre for Antibiotic resistance (ANRESIS).

*: Prescription of Fosfomycin, Nitrofurantoin or TMP/SMX were considered as recommended therapy.

Supplemental table 2a – Logistic regression analysis for antibiotics prescriptions.

|  |  | **Prescription of guideline recommended antibiotic*** | | | |  | **Prescription of fluoroquinolone antibiotic^+^** | | | |
| --- | --- | --- | --- | --- | --- | --- | --- | --- | --- | --- |
|  |  | **Univariable analysis** | | **Multivariable analysis N=1133, GP=152, ICC=0.34** | |  | **Univariable analysis** | | **Multivariable analysis N=1090, GP=151, ICC=0.41** | |
| **GP characteristics** | **N, N of GP** | **OR (95% CI)** | **p-value** | **OR (CI 95%)** | **p-value** | **N, GP** | **OR (95% CI)** | **p-value** | **OR (95% CI)** | **p-value** |
| Sex of GP = female | 1192, 158 | 1.05 (0.60, 1.83) | 0.858 |  |  | 1167, 158 | 0.99 (0.54, 1.81) | 0.974 |  |  |
| Age of GP | 1141, 152 | **0.97 (0.94, 0.99)** | **0.019** | **0.97 (0.93, 1.00)** | **0.049** | **1116, 152** | **1.03 (1.00, 1.07)** | **0.047** | **1.04 (1.00, 1.08)** | **0.033** |
| Years of GP experience in practice | 1106, 147 | 0.97 (0.95, 1) | 0.062 |  |  | 1083,147 | 1.02 (0.99, 1.05) | 0.187 |  |  |
| Practice type  (ref = Single)  Double  Group | 1174, 156 | 1.94 (0.96, 3.91)  1.63 (0.90, 2.97) | 0.065  0.107 |  |  | 1149, 156 | 0.47 (0.22, 1.02)  0.56 (0.29, 1.08) | 0.057  0.084 |  |  |
| Work time%  (ref = 30-60%)  70-80 %  85-100 % | 1166, 155 | 1.14 (0.53, 2.45)  0.58 (0.31, 1.10) | 0.733  0.098 |  |  | 1141, 155 | 0.91 (0.39, 2.11)  1.72 (0.85, 3.48) | 0.828  0.128 |  |  |
| Affiliated to a medical network | 1174, 156 | 1.20 (0.69, 2.06) | 0.522 |  |  | 1149, 156 | 0.94 (0.51, 1.72) | 0.831 |  |  |
| Language of GP  (ref = German)  Latin (Italian and French) | 1192, 158 | 1.09 (0.52, 2.28) | 0.820 |  |  | 1167, 158 | 0.87 (0.38, 1.96) | 0.731 |  |  |
| Location of GP (ref=Other)  Region lemanique  Ticino  Zürich | 1192, 158 | 2.69 (0.93, 7.83)  0.32 (0.06, 1.53)  1.89 (0.90, 3.96) | 0.069  0.153  0.092 | 2.35 (0.71, 7.77)  0.33 (0.05, 2.03)  1.87 (0.77, 4.55) | 0.160  0.230  0.166 | 1167, 158 | 0.37 (0.12, 1.19)  3.56 (0.64, 19.8)  0.54 (0.24, 1.21) | 0.095  0.147  0.134 |  |  |
| **Patient characteristics** | **N** |  | | | | **N** |  | | | |
| Previous urinary tract infection | **1240** | **1.7 (1.20, 2.37)** | **0.002** |  |  | 1216 | **0.55 (0.39, 0.79)** | **0.001** | 0.71 (0.43, 1.17) | 0.181 |
| Antibiotics in the last 3 months | 1235 | 0.80 (0.59, 1.11) | 0.177 |  |  | 1211 | 1.27 (0.91, 1.75) | 0.157 | 1.42 (0.91, 2.22) | 0.121 |
| Inpatient treatment in the last 6 months | 1241 | 0.78 (0.51, 1.24) | 0.283 |  |  | 1216 | 1.21 (0.75, 1.90) | 0.421 |  |  |
| Travel history within the last 12 months | 1224 | **1.40 (1.05, 1.87)** | **0.020** |  |  | 1200 | **0.70 (0.52, 0.95)** | **0.020** |  |  |
| Travel within Europe | 1224 | **1.46 (1.10, 1.95)** | **0.009** |  |  | 1200 | **0.73 (0.54, 0.98)** | **0.035** |  |  |
| Travel to Asia | 1224 | 0.92 (0.55, 1.62) | 0.756 |  |  | 1200 | 1.07 (0.59, 1.84) | 0.813 |  |  |
| Travel to Africa | 1224 | 0.89 (0.44, 2.01) | 0.769 |  |  | 1200 | 1.10 (0.47, 2.30) | 0.811 |  |  |
| Travel to Nord-America | 1224 | 1.09 (0.54, 2.41) | 0.826 |  |  | 1200 | 1.02 (0.46, 2.05) | 0.950 |  |  |
| Travel to South-America | 1224 | 0.73 (0.35, 1.66) | 0.423 |  |  | 1200 | 1.00 (0.37, 2.28) | 0.998 |  |  |
| Diagnosis Complicated UTI | **1241** | **0.14 (0.09, 0.20)** | **<0.001** | **0.12 (0.05, 0.27)** | **<0.001** | **1216** | **7.32 (4.9 , 11.1)** | **<0.001** | **8.95 (3.62, 22.10)** | **<0.001** |
| Sex of Patient = male | **1249** | **0.10 (0.06, 0.17)** | **<0.001** | **0.36 (0.13, 0.99)** | **0.048** | **1224** | **9.9 (5.74, 17.5)** | **<0.001** | 3.13 (0.99, 9.87) | 0.052 |
| Initial encounter not because symptoms suspicious for an UTI | 1240 | 1.16 (0.67, 2.13) | 0.618 |  |  | 1215 | 0.69 (0.34, 1.28) | 0.274 |  |  |

Supplemental table 2b: Regression analysis identifying GP and patient characteristics as predictor for prescribing any quinolone antibiotic^+^ (vs. recommended)

|  | **Uncomplicated UTI** | | | | | **Complicated UTI** | | | | |
| --- | --- | --- | --- | --- | --- | --- | --- | --- | --- | --- |
|  | **Univariable analysis** | | | **Multivariable analysis N=995, GP=149, ICC=0.39** | | **Univariable analysis** | | | **Multivariable analysis N=110, GP=67, ICC=0.32** | |
|  | **N** | **OR (95% CI)** | **p** | **OR (95% CI)** | **p** | **N** | **OR (95% CI)** | **p** | **OR (95% CI)** | **p** |
| **GP Characteristics** | | | | | | | | | | |
| Sex of GP = female | N=1049, GP=156 | 0.76 (0.36, 1.6) | 0.473 |  |  | N=110, GP=67 | **5.68 (1.31, 24.6)** | **0.020** | **7.08 (1.66, 30.10)** | **0.008** |
| Age | N=1005, GP=150 | **1.06 (1.02 , 1.1)** | **0.003** | **1.06 (1.02, 1.10)** | **0.003** | N=103, GP=63 | 0.97 (0.91, 1.04 ) | 0.444 |  |  |
| Years of experience in GP practice | N=975, GP=146 | 1.03 (1.00, 1.07) | 0.063 |  |  | N=100, GP=59 | 1.01 (0.93, 1.10) | 0.766 |  |  |
| Practice type (ref =Single)  Double  Group | N=1034, GP=154 | **0.40 (0.16 , 0.98)**  **0.40 (0.18 , 0.86)** | **0.046**  **0.019** |  |  | N=107, GP=65 | 0.60 (0.09, 3.78)  1.56 (0.33, 7.32) | 0.587  0.575 |  |  |
| Work-time% (ref = 30-60%)  70-80 %  85-100 % | N=1028, GP=153 | 1.02 (0.37, 2.79)  **2.46 (1.06, 5.74)** | 0.966  **0.037** |  |  | N=105, GP=64 | 4.54 (0.62 , 33.2)  1.03 (0.27, 3.97) | 0.136  0.962 |  |  |
| Affiliated to a MNW | N=1034, GP=154 | 0.85 (0.41, 1.73) | 0.650 |  |  | N=107, GP=65 | 1.93 (0.45, 8.28) | 0.379 |  |  |
| Language of GP  (ref German)  Latin | N=1049, GP=156 | 0.85 (0.32, 2.26) | 0.752 |  |  | N=110, GP=67 | 0.44 (0.06, 3.30) | 0.427 |  |  |
| Location of GP (ref=Other)  Region lemanique  Ticino  Zürich | N=1049, GP=156 | 0.45 (0.12, 1.68)  3.96 (0.55, 28.7)  **0.33 (0.12, 0.95)** | 0.235  0.173  **0.039** |  |  | N=110, GP=67 | 0.15 (0.00, 7.23)  -  0.68 (0.13, 3.66) | 0.340  -  0.658 |  |  |
| **Patient characteristics** | | | | | | | | | | |
| History of UTI | N=1096 | 0.75 (0.49, 1.17) | 0.194 |  |  | N=112 | **0.35 (0.14, 0.81)** | **0.017** | **0.26 (0.08, 0.83)** | **0.023** |
| Antibiotic exposure within the last 3 months | N=1094 | 1.42 (0.97, 2.05) | 0.067 | 1.51 (0.94, 2.40) | 0.086 | N=109 | **0.43 (0.19, 0.97)** | **0.044** |  |  |
| Inpatient treatment within the last 6 months | N=1099 | 1.26 (0.71, 2.11) | 0.404 |  |  | N=109 | 0.76 (0.24, 2.38) | 0.631 |  |  |
| Travel history within the last 12 months | N=1082 | **0.62 (0.44, 0.88)** | **0.007** |  |  | N=111 | 1.03 (0.48, 2.21) | 0.944 |  |  |
| Sex of Patient = male | - | **-** | **-** |  |  | N=112 | **2.36 (1.11 , 5.11)** | **0.027** |  |  |
| Initial encounter not because symptoms suspicious for an UTI | N=1097 | 0.72 (0.31, 1.44) | 0.386 |  |  | N=110 | 0.63 (0.12, 2.99) | 0.557 |  |  |

Supplemental table 2c: Regression analysis identifying GP and patient characteristics affecting GP antibiotic prescribing patterns

|  | **TMP/SMX** | | | | | **Fosfomycin** | | | | |
| --- | --- | --- | --- | --- | --- | --- | --- | --- | --- | --- |
|  | **Univariable analysis** | | | **Multivariable analysis N=1150, GP=151, ICC=0.34** | | **Univariable analysis** | | | **Multivariable analysis N=1205, GP=154, ICC=0.39** | |
|  | **N** | OR (95% CI) | p | OR (95% CI) | p | **N** | OR (95% CI) | p | OR (95% CI) | p |
| **GP Characteristics** | | | | | | | | | | |
| Sex of GP = female | N=1192, GP=158 | 0.81 (0.45, 1.46) | 0.484 |  |  | N=1192, GP=158 | 1.24 (0.72, 2.12) | 0.442 |  |  |
| Age | N=1141, GP=152 | 1.01 (0.98, 1.05) | 0.389 |  |  | N=1141, GP=152 | 0.99 (0.97, 1.02) | 0.742 |  |  |
| Years of experience in GP practice | N=1106, GP=147 | 1 (0.99, 1.00) | 0.328 |  |  | N=1106, GP=147 | 0.99 (0.96, 1.02) | 0.600 |  |  |
| Practice type (ref =Single)  Double  Group | N=1174, GP=156 | 1.07 (0.51, 2.2)  0.54 (0.28, 1.04) | 0.858  0.064 | 1.05 (0.49, 2.22)  0.53 (0.27, 1.04) | 0.906  0.066 | N=1174, GP=156 | 1.39 (0.69, 2.82)  1.38 (0.74, 2.56) | 0.358  0.306 |  |  |
| Work-time%  (ref = 30-60%)  70-80 %  85-100 % | N=1166 GP=155 | 0.72 (0.33, 1.60)  1.28 (0.66, 2.49) | 0.423  0.461 |  |  | N=1166, GP=155 | 1.00 (0.48, 2.07)  0.72 (0.39, 1.34) | 0.998  0.302 |  |  |
| Affiliated to a MNW | N=1174, GP=156 | 0.75 (0.42, 1.35) | 0.344 |  |  | N=1174, GP=156 | 1.20 (0.69, 2.06) | 0.519 |  |  |
| Language of GP  (ref = German)  Latin | N=1192, GP=158 | 1.18 (0.55, 2.50) | 0.669 |  |  | N=1192,  GP=158 | 0.68 (0.33, 1.38) | 0.285 |  |  |
| Location of GP (ref=Other)  Region lemanique  Ticino  Zürich | N=1192, GP=158 | 1.01 (0.37, 2.73)  1.10 (0.19, 6.50)  0.57 (0.26, 1.23) | 0.982  0.916  0.150 |  |  | N=1192, GP=158 | 1.08 (0.43 , 2.69)  0.54 (0.09, 3.1)  1.69 (0.84, 3.39) | 0.873  0.487  0.139 |  |  |
| **Patient characteristics** | | | | | | | | | | |
| History of UTI | N=1240 | 1.15 (0.82, 1.64) | 0.424 |  |  | N=1240 | 1.01 (0.75, 1.36) | 0.955 |  |  |
| Antibiotic exposure within the last 3 months | N=1235 | 0.91 (0.67, 1.22) | 0.541 |  |  | N=1235 | **0.62 (0.48, 0.81)** | **0.001** | **0.60 (0.43, 0.83)** | **0.002** |
| Inpatient treatment within the last 6 months | N=1241 | 0.98 (0.63, 1.49) | 0.935 |  |  | N=1241 | 0.73 (0.49, 1.06) | 0.107 |  |  |
| Travel history within the last 12 months | N=1224 | **0.74 (0.57, 0.96)** | **0.024** | 0.77 (0.56, 1.07) | 0.123 | N=1224 | **1.46 (1.16, 1.85)** | **0.001** | **1.50 (1.11, 2.05)** | **0.009** |
| Final diagnosis complicated | N=1241 | **0.51 (0.30, 0.84)** | **0.012** |  |  | N=1241 | **0.21 (0.12, 0.34)** | **<0.001** | **0.15 (0.06, 0.36)** | **<0.001** |
| Sex of patient = male | N=1249 | 0.77 (0.40, 1.40) | 0.423 |  |  | N=1249 | **0.11 (0.04 , 0.26)** | **<0.001** | 0.29 (0.07, 1.14) | 0.077 |
| Initial encounter not because symptoms suspicious for an UTI | N=1240 | 0.90 (0.52, 1.48) | 0.692 |  |  | N=1240 | 1.15 ( 0.74 , 1.78) | 0.539 |  |  |

|  | **Nitrofurantoin** | | | | | **Ciprofloxacin** | | | | |
| --- | --- | --- | --- | --- | --- | --- | --- | --- | --- | --- |
|  | **Univariable analysis** | | | **Multivariable analysis N=1121, GP=151, ICC=0.61** | | **Univariable analysis** | | | **Multivariable analysis N=1143, GP=151, ICC=0.45** | |
|  | **N** | OR (95% CI) | p | OR (95% CI) | p | **N** | OR (95% CI) | p | OR (95% CI) | p |
| **GP Characteristics** | | | | | | | | | | |
| Sex of GP = female | N=1192, GP=158 | 1.36 (0.49, 3.77) | 0.556 |  |  | N=1192, GP=158 | 0.90 (0.46, 1.74) | 0.751 |  |  |
| Age | N=1141, GP=152 | **0.92 (0.87, 0.97)** | **0.002** | **0.91 (0.86, 0.96)** | **0.001** | N=1141, GP=152 | 1.00 (0.96, 1.03) | 0.968 |  |  |
| Years of experience in GP practice | N=1106, GP=147 | **0.94 (0.89, 0.99)** | **0.032** |  |  | N=1106, GP=147 | 1.00 (0.97, 1.04) | 0.804 |  |  |
| Practice type (ref =Single)  Double  Group | N=1174, GP=156 | 1.68 (0.43, 6.59)  **4.41 (1.33, 14.60)** | 0.460  **0.015** |  |  | N=1174, GP=156 | 0.51 (0.22, 1.17)  0.56 (0.28, 1.12) | 0.111  0.098 |  |  |
| Work-time%  (ref = 30-60%)  70-80 %  85-100 % | N=1166, GP=155 | 1.83 (0.50, 6.72)  0.36 (0.11, 1.16) | 0.362  0.088 |  |  | N=1166, GP=155 | 0.60 (0.23, 1.55)  1.43 (0.68, 3.00) | 0.294  0.346 |  |  |
| Affiliated to a MNW | N=1174, GP=156 | 1.30 (0.46, 3.67) | 0.620 |  |  | N=1174, GP=156 | **0.49 (0.26, 0.91)** | **0.024** | 0.43 (0.17, 1.09) | 0.076 |
| Language of GP  (ref=German)  Latin | N=1192, GP=158 | 2.51 (0.70, 8.97) | 0.155 |  |  | N=1192, GP=158 | 1.13 (0.47, 2.68) | 0.784 |  |  |
| Location of GP (ref=Other)  Region lemanique  Ticino  Zürich | - | - | - |  |  | N=1192, GP=158 | 0.43 (0.11, 1.61)  **7.50 (1.46, 38.6)**  0.42 (0.16, 1.09) | 0.208  **0.016**  0.074 | 0.29 (0.05, 1.79)  -  0.35 (0.09, 1.29) | 0.182  -  0.115 |
| **Patient characteristics** | | | | | | | | | | |
| History of UTI | N=1240 | **1.86 (1.17, 3.12)** | **0.013** | 1.59 (0.79, 3.21) | 0.198 | N=1240 | **0.53 (0.35, 0.82)** | **0.004** |  |  |
| Antibiotic exposure within the last 3 months | N=1235 | **2.13 (1.52, 2.96)** | **<0.001** | **2.24 (1.37, 3.65)** | **0.001** | N=1235 | 1.43 (0.94, 2.13) | 0.089 |  |  |
| Inpatient treatment within the last 6 months | N=1241 | 1.31 (0.78, 2.10) | 0.280 |  |  | N=1241 | 1.07 (0.56, 1.90) | 0.821 |  |  |
| Travel history within the last 12 months | N=1224 | 1.11 (0.80, 1.54) | 0.542 |  |  | N=1224 | **0.64 (0.44, 0.94)** | **0.024** | 0.62 (0.36, 1.05) | 0.075 |
| Final diagnosis complicated | N=1241 | 0.91 (0.51, 1.55) | 0.749 |  |  | N=1241 | **12.6 (8.17, 19.6)** | **<0.001** | **28.26 (10.23, 78.04)** | **<0.001** |
| Sex of patient =male | N=1249 | 0.28 (0.07, 0.78) | 0.036 | **0.18 (0.04, 0.90)** | **0.037** | N=1249 | **13.7 (8.00, 23.7)** | **<0.001** | 2.87 (0.90, 9.13) | 0.074 |
| Initial encounter not because symptoms suspicious for an UTI | N=1240 | 1.04 (0.54, 1.85) | 0.907 |  |  | N=1240 | 0.96 (0.42 1.92) | 0.916 |  |  |

*: consist of a summary group of fosfomycin, TMP-SMX, and nitrofurantoin. ^+^: consist a summary of ciprofloxacin, levofloxacin, moxifloxacin, and norfloxacin

GP: general practitioner; MNW: medical network; UTI: urinary tract infection; OR: odds ratio; 95% CI: 95% confidence interval, ref: reference; f: female; TMP/SMX: trimethoprim-sulfamethoxazol;

Supplemental table 2d: Logistic regression analysis for no antibiotics prescription.

|  |  | **Prescribing no antibiotics**  **(outcome 1= no antibiotics, 0 = any AB)** | | | |  | **Prescribing no antibiotics**  **(outcome 1= no antibiotics, 0 = recommended AB)** | | | |
| --- | --- | --- | --- | --- | --- | --- | --- | --- | --- | --- |
|  |  | **Univariable analysis** | | **Multivariable analysis N=1229, GP=155, ICC=0.32** | |  | **Univariable analysis** | | **Multivariable analysis N=1002, GP=147, ICC=0.30** | |
| **GP Characteristics** | **N** | **OR CI (95%)** | **p** | **OR CI (95%)** | **p** | **N** | **OR CI (95%)** | **p** | **OR CI (95%)** | **p** |
| Sex | N=1295,  GP=161 | 0.97 (0.50, 1.87) | 0.928 |  |  | N=1056,  GP=153 | 0.93 (0.48, 1.81) | 0.828 |  |  |
| Age | N=1239,  GP=155 | 1.03 (0.99, 1.07) | 0.100 | 1.03 (1.00, 1.07) | 0.083 | N=1009,  GP=147 | **1.04 (1, 1.08)** | **0.037** | **1.04 (1.00, 1.08)** | **0.036** |
| Years of GP experience in practice | N=1196,  GP=150 | 1.01 (0.98, 1.04) | 0.532 |  |  | N=968,  GP=142 | 1.02 (0.98, 1.05) | 0.314 |  |  |
| Practice type (ref = Single)  Double  Group | N=1276,  GP=159 | 1.07 (0.46, 2.47)  0.88 (0.42, 1.84) | 0.876  0.737 |  |  | N=1038,  GP=151 | 0.94 (0.40, 2.20) 0.78 (0.37, 1.66) | 0.883  0.527 |  |  |
| Work-time%  (ref 30-60%)  70-80 %  85-100 % | N=1268,  GP=158 | 0.95 (0.40, 2.26)  0.84 (0.40, 1.75) | 0.910  0.634 |  |  | N=1032,  GP=150 | 0.93 (0.38, 2.24) 0.96 (0.45, 2.03) | 0.865  0.906 |  |  |
| Affiliated to a medical network | N=1276,  GP=159 | 0.59 (0.32, 1.11) | 0.102 |  |  | N=1038,  GP=151 | 0.57 (0.30, 1.07) | 0.082 |  |  |
| Language of GP (ref=German)  Latin | N=1295, GP=161 | 0.62 (0.24, 1.61) | 0.328 |  |  | N=1056,  GP=153 | 0.61 (0.23, 1.61) | 0.317 |  |  |
| Location of GP (ref=Other)  Region lemanique  Ticino  Zürich | N=1295, GP=161 | 0.77 (0.22, 2.66)  1.36 (0.18, 10.3)  1.50 (0.68, 3.34) | 0.676  0.769  0.317 |  |  | N=1056,  GP=153 | 0.66 (0.19, 2.32)  1.90 (0.22, 16.7)  1.35 (0.60, 3.03) | 0.521  0.562  0.470 |  |  |
| Complicated UTI | N=1339 | 1.07 (0.51, 2.02) | 0.843 |  |  | N=1099 | **2.12 (0.98 , 4.15)** | **0.039** |  |  |
| Sex of Patient = female) | N=1352 | 1.16 (0.44, 2.55) | 0.729 |  |  | N=1110 | **2.90 (1.05, 6.96)** | **0.025** | 2.99 (0.96, 9.34) | 0.059 |
| Reason for encounter: Other than UTI | N=1342 | **2.84 (1.59, 4.84)** | **<0.001** | **3.37 (1.73, 6.56)** | **<0.001** | N=1103 | **2.76 (1.54 , 4.76)** | **<0.001** | **2.97 (1.49, 5.91)** | **0.002** |
